# Supplementary material for: Simulation of pH-Dependent Conformational Transitions in Membrane Proteins: The CLC-ec1 Cl−/H+ Antiporter
Source: Molecules. 2021 Nov 18;26(22):6956. doi: 10.3390/molecules26226956 (PMC8625536; doi:10.3390/molecules26226956)
Supplement: Supplementary file 1 [file molecules-26-06956-s001.zip › molecules-1458565-supplementary.pptx]

## Slide 1
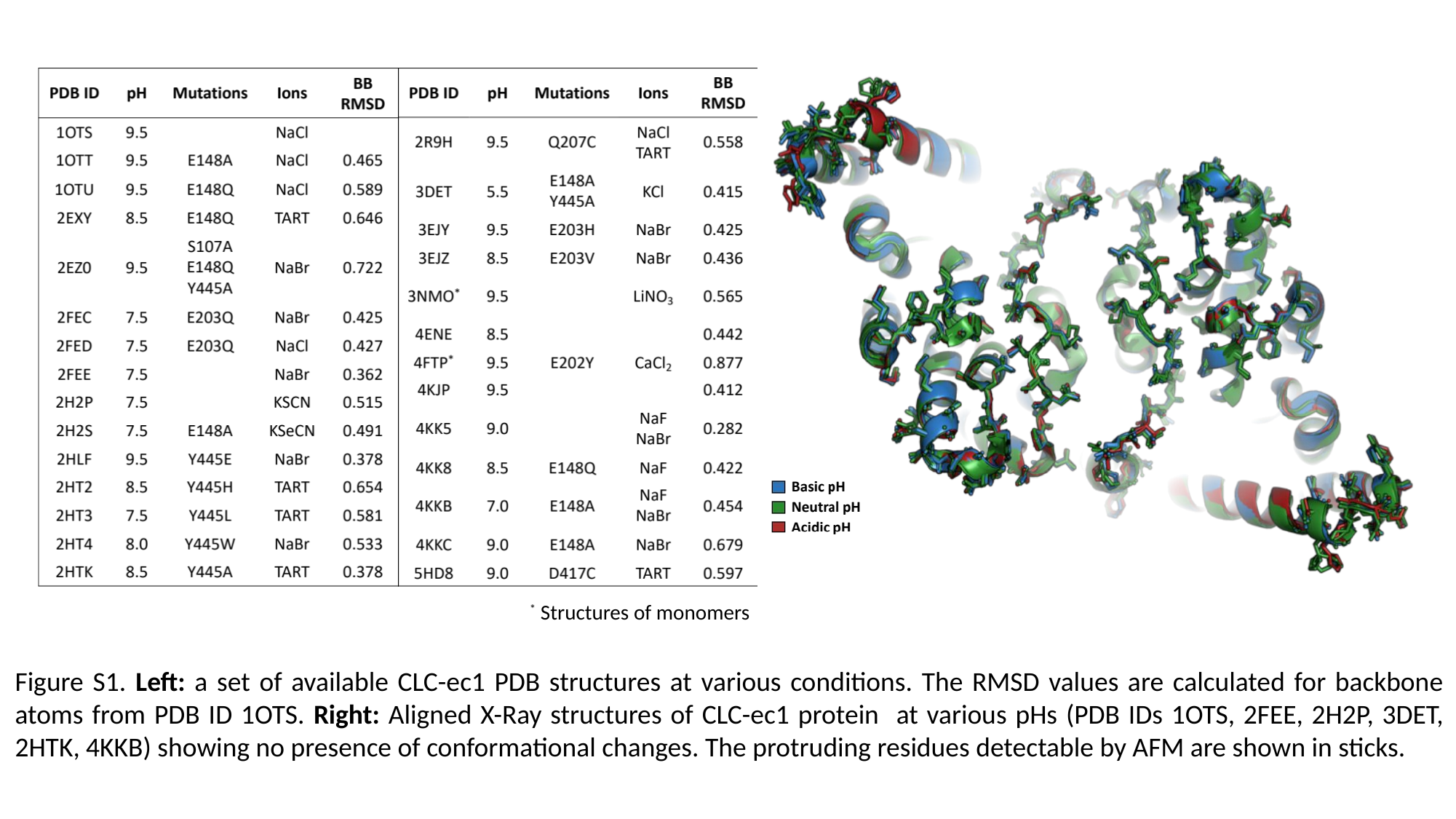

* Structures of monomers
Figure S1. Left: a set of available CLC-ec1 PDB structures at various conditions. The RMSD values are calculated for backbone atoms from PDB ID 1OTS. Right: Aligned X-Ray structures of CLC-ec1 protein at various pHs (PDB IDs 1OTS, 2FEE, 2H2P, 3DET, 2HTK, 4KKB) showing no presence of conformational changes. The protruding residues detectable by AFM are shown in sticks.

## Slide 2
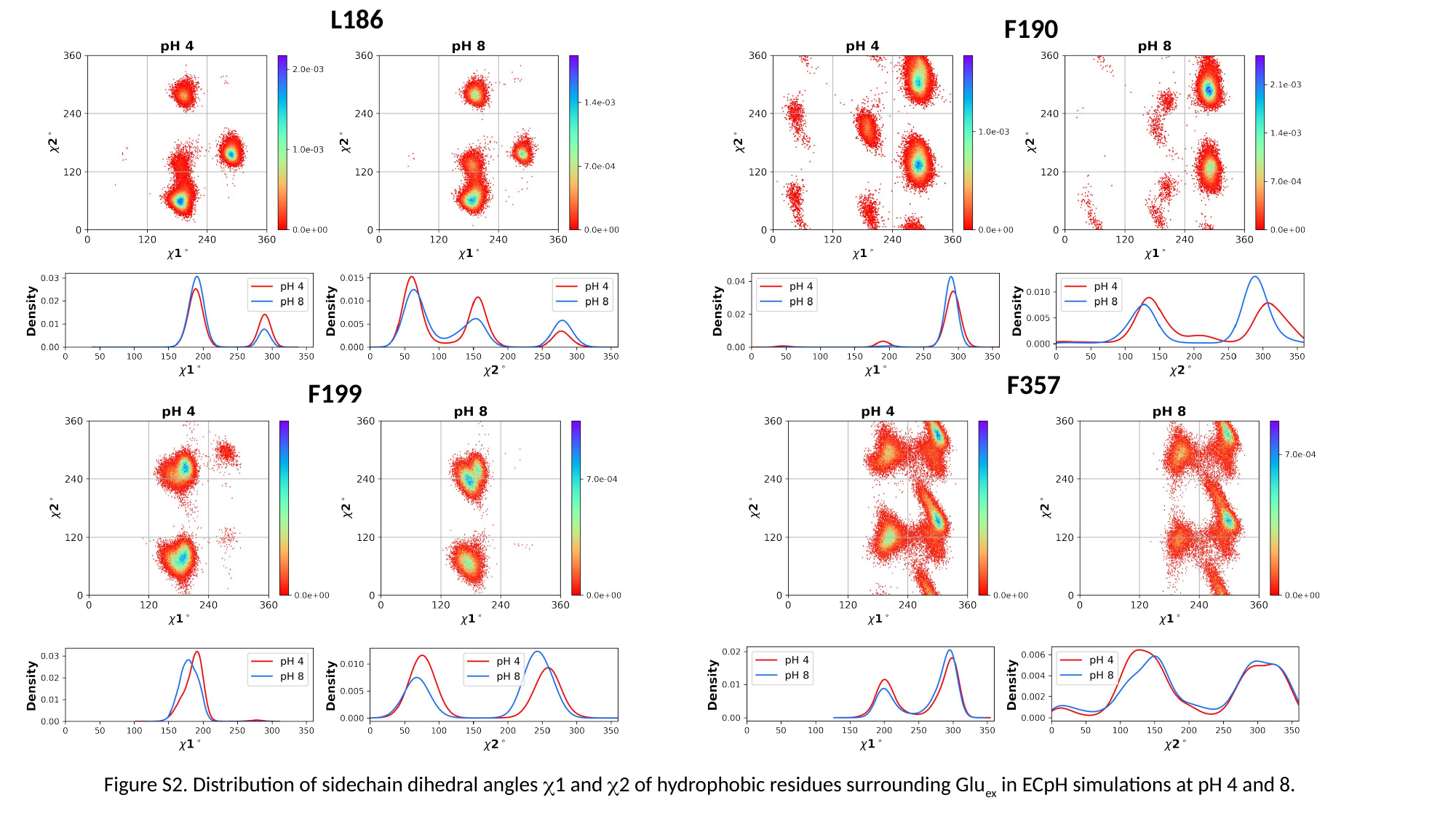

L186
F190
F357
F199
Figure S2. Distribution of sidechain dihedral angles c1 and c2 of hydrophobic residues surrounding Gluex in ECpH simulations at pH 4 and 8.

## Slide 3
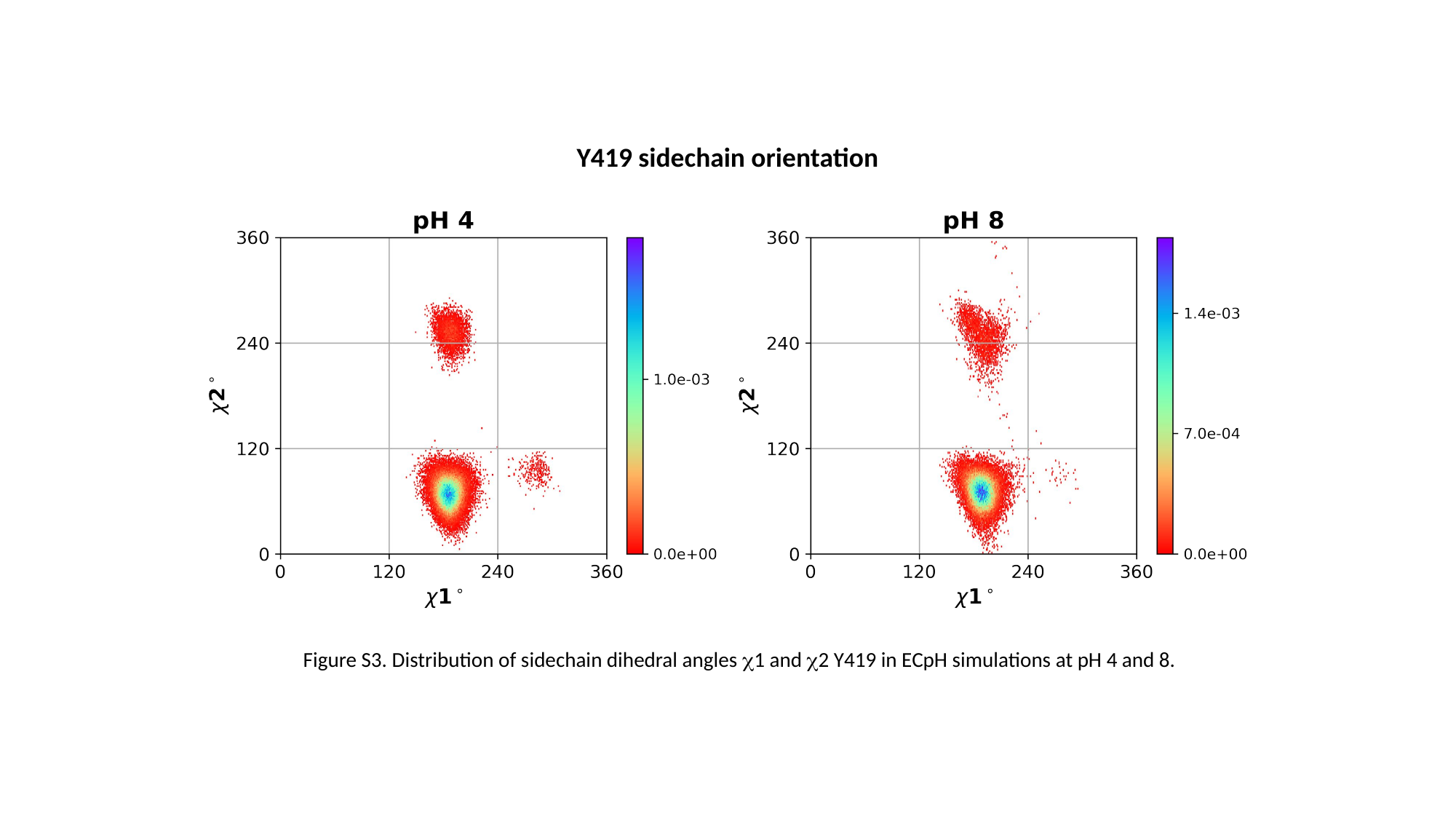

Y419 sidechain orientation
Figure S3. Distribution of sidechain dihedral angles c1 and c2 Y419 in ECpH simulations at pH 4 and 8.

## Slide 4
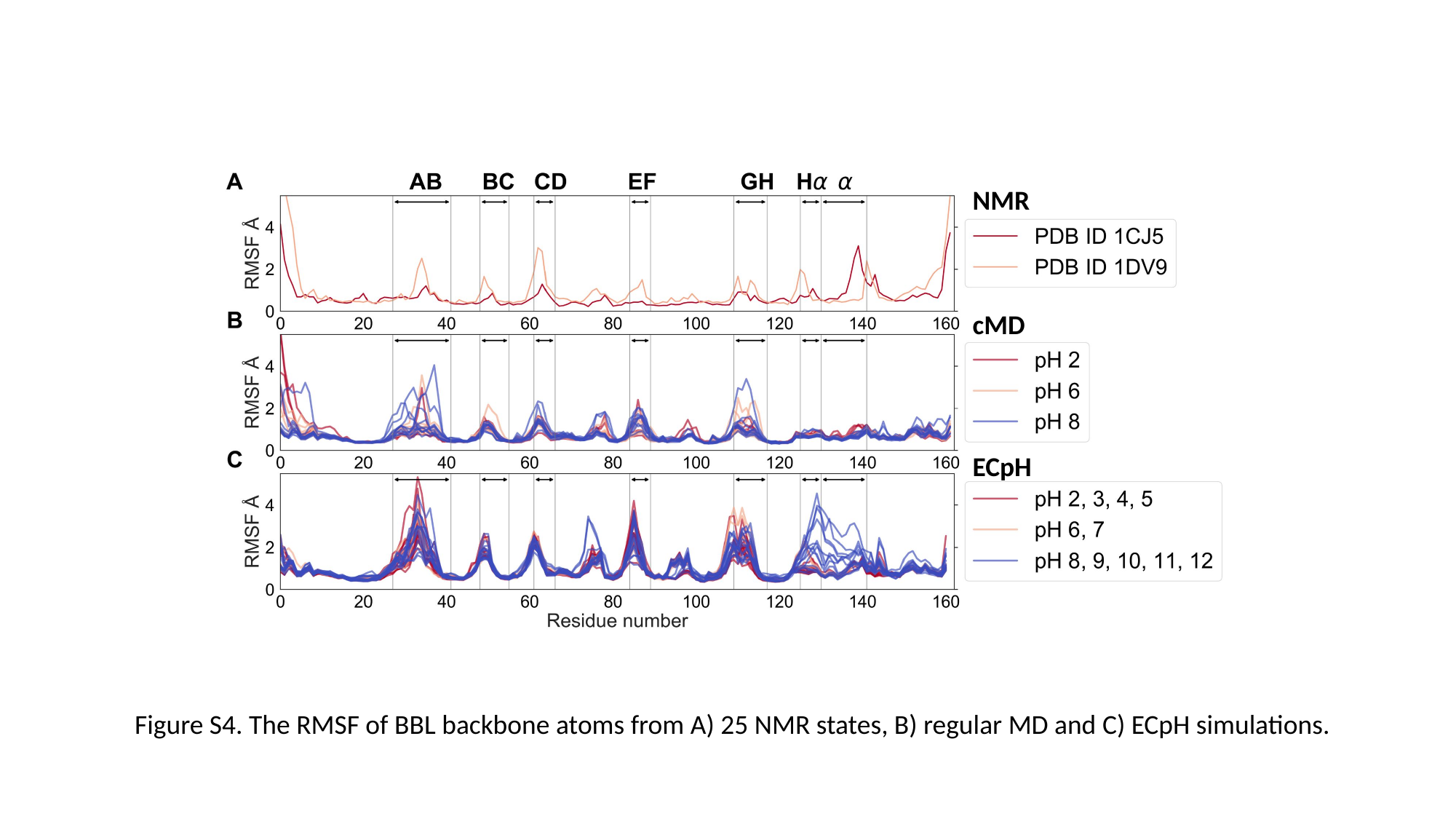

NMR
cMD
ECpH
Figure S4. The RMSF of BBL backbone atoms from A) 25 NMR states, B) regular MD and C) ECpH simulations.

## Slide 5
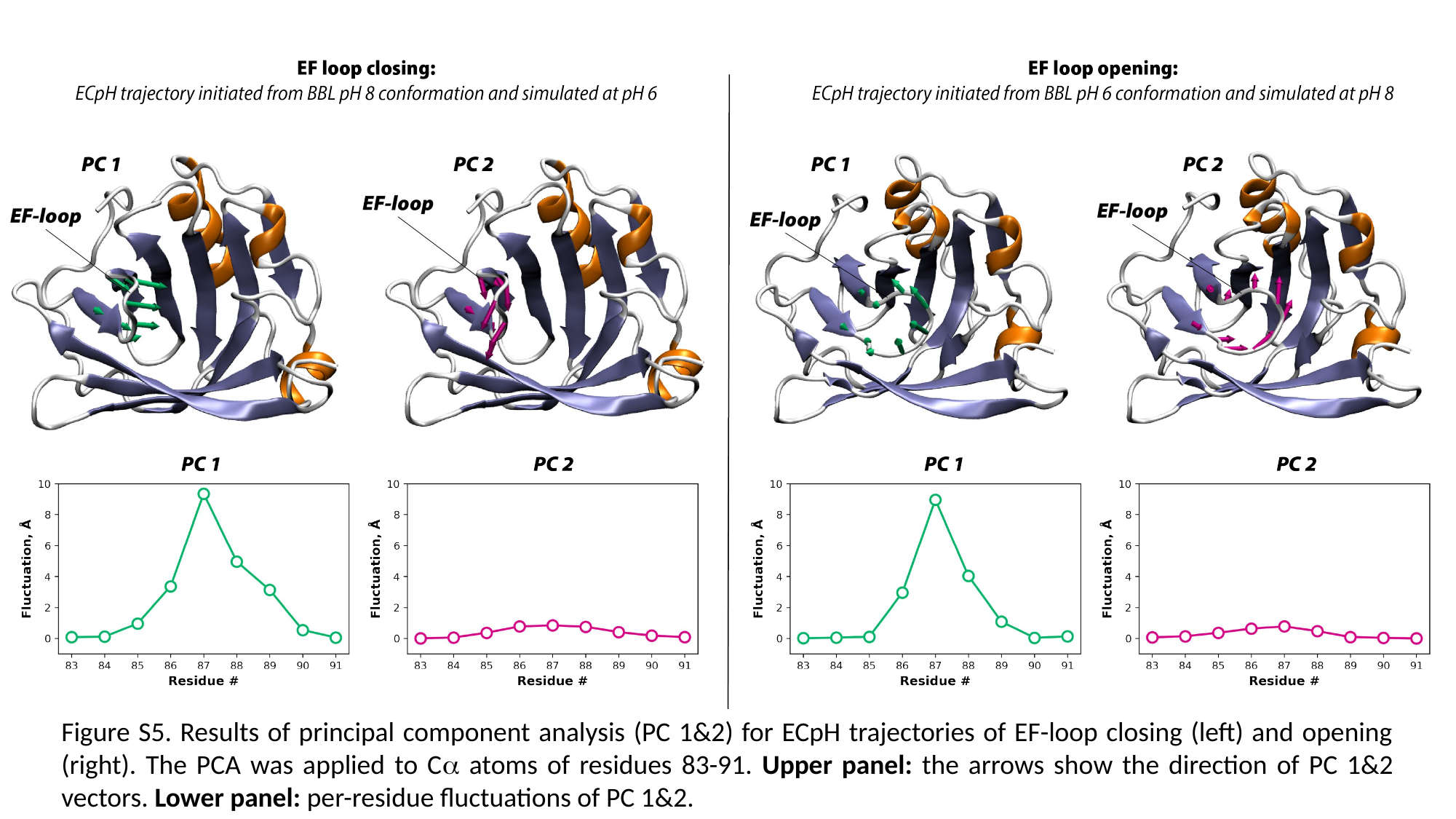

Figure S5. Results of principal component analysis (PC 1&2) for ECpH trajectories of EF-loop closing (left) and opening (right). The PCA was applied to Ca atoms of residues 83-91. Upper panel: the arrows show the direction of PC 1&2 vectors. Lower panel: per-residue fluctuations of PC 1&2.

## Slide 6
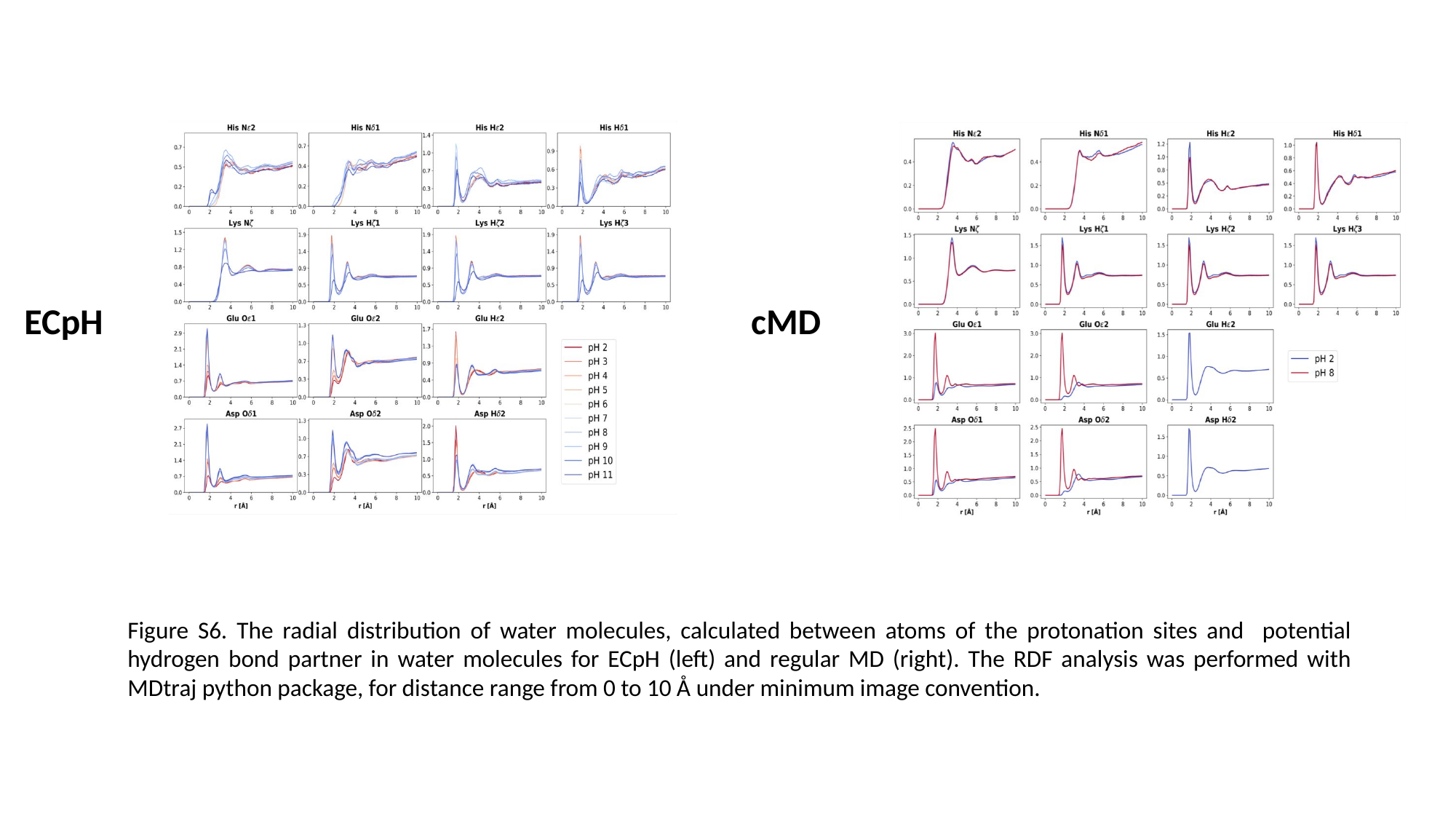

ECpH
cMD
Figure S6. The radial distribution of water molecules, calculated between atoms of the protonation sites and potential hydrogen bond partner in water molecules for ECpH (left) and regular MD (right). The RDF analysis was performed with MDtraj python package, for distance range from 0 to 10 Å under minimum image convention.
